# Exploration into the MLL4/WRAD Enzyme-Substrate Network: Systematic In Vitro Identification of CFP1 as a Potential Non-Histone Substrate of the MLL4 Lysine Methyltransferase

**DOI:** 10.3390/epigenomes9040041

**Published:** 2025-10-15

**Authors:** Mullen Boulter, Ryan Collins, Kyle K. Biggar

**Affiliations:** Institute of Biochemistry, Carleton University, Ottawa, ON K1S 5B6, Canada

**Keywords:** non-histone methylation, MLL4, KMT2D, CFP1, lysine methylation, methyl-switch

## Abstract

Lysine methylation is a critical post-translational modification catalyzed by lysine methyltransferases (KMTs), originally characterized in the regulation of histones. However, the breadth of non-histone targets remains largely unexplored. Here, we used a systematic peptide array-based approach to define a substrate preference motif for the SET-domain-containing KMT MLL4 (KMT2D), a member of the COMPASS complex and a known H3K4 methyltransferase. Using this motif, we identified CXXC finger protein 1 (CFP1), a core component of Setd1A/B complexes, as a putative MLL4 substrate. In vitro methyltransferase assays confirmed robust methylation of CFP1 by an MLL4-WRAD complex. Surprisingly, while initial predictions implicated K328, array-based methylation profiling revealed multiple lysine residues within CFP1’s lysine-rich basic domain as methylation targets, including K331, K335, K339, and K340. We further demonstrated that CFP1 methylation likely modulates its interaction with MLL4’s PHD cassettes and facilitates binding to Setd1A. Binding preferences of MLL4’s PHD1–3 and PHD4–6 domains varied with methylation state and site, suggesting non-histone methyl mark recognition by these cassettes. Pulldown assays confirmed that methylated, but not unmethylated, CFP1 binds Setd1A, supporting a potential methyl-switch mechanism. Together, our findings propose CFP1 as a potential non-histone substrate of MLL4 and suggest that MLL4 may regulate Setd1A/B function indirectly via CFP1 methylation. This study expands the substrate landscape of MLL4 and lays the groundwork for future investigations into non-histone methylation signaling in chromatin regulation.

## 1. Introduction

Protein methylation is a critical post-translational modification (PTM) involved in regulating diverse biological processes. Methylation can occur on several amino acid residues, including histidine, arginine, and lysine. Lysine methylation was initially thought to be restricted to histone substrates, supporting the histone code hypothesis (reviewed in [1,2,3]). However, growing evidence suggests that lysine methylation also extends to non-histone proteins, implicating this modification in pathways beyond gene regulation (reviewed in [4]). Lysine methylation is catalyzed by lysine methyltransferases (KMTs), which transfer a methyl group from the donor molecule S-adenosyl methionine (SAM) to lysine residues on target proteins.

Structurally, KMTs are categorized into two main classes: the 7 β-strand (7βS) KMTs and the SET domain-containing KMTs (Suppressor of variegation, Enhancer of zeste, and Trithorax). To date, approximately 120 7βS KMTs [5] and more than 55 SET-domain KMTs [6] have been identified, underscoring the extensive regulatory potential of lysine methylation. This process is reversible, with lysine demethylases (KDMs) removing methyl groups. Lysine residues can be mono-, di-, or tri-methylated depending on the enzymatic context and substrate specificity, adding another layer of regulation. SET domain KMTs primarily catalyze histone methylation and are classified based on their histone targets [7]. One such enzyme, MLL4 (also known as KMT2D or ALR), is a member of the KMT2 family that methylates histone H3 at lysine 4 (H3K4). H3K4 methylation is a conserved event, mediated by Set1 in *Saccharomyces cerevisiae* [7] and by dSet1, Trithorax, Trithorax-related, and Ash1 in *Drosophila melanogaster* [8]. Studies in these models have highlighted the essential roles of KMT2 enzymes in processes such as embryonic proliferation [9,10,11,12].

Mammals possess six Set1 paralogs: Setd1A, Setd1B, MLL1, MLL2, MLL3, and MLL4. These enzymes share overlapping and unique roles in H3K4 methylation and beyond [13,14,15,16]. Their activity is regulated through a multimeric complex known as COMPASS (COMplex of Proteins ASsociated with Set1), which includes four core subunits: Ash2L, RbBP5, WDR5, and Dpy-30, collectively referred to as the WRAD complex [7]. The WRAD complex facilitates recruitment and activation of Set1/MLL proteins, and its components serve non-redundant functions critical to global H3K4 methylation [17,18,19]. Additionally, accessory proteins like CFP1 (CXXC finger protein 1), menin, and UTX associate with distinct COMPASS subcomplexes, further tuning functional specificity [7].

MLL (mixed-lineage leukemia) family members gained prominence after chromosomal translocations at 11q23 were linked to poor prognosis in certain leukemia subtypes. This region encodes a homolog of *Drosophila* trithorax, containing the conserved SET domain responsible for H3K4 methylation [20,21]. MLL1 and MLL2 are trithorax descendants, while MLL3 and MLL4 derive from trithorax-related genes. This evolutionary divergence correlates with substrate specificity: MLL1/2 primarily catalyze mono- and di-methylation, whereas MLL3/4 are largely mono-methyltransferases [22]. Although all MLLs regulate homeotic gene expression, their divergent SET domains suggest distinct, possibly novel, substrate repertoires—particularly among non-histone proteins. MLL4, a mono-H3K4 methyltransferase, functions through its SET domain in association with the WRAD complex, enhancing gene expression in a cell-type-specific manner [23]. While closely related to MLL3, MLL4 has essential non-redundant roles [24]. Structurally, MLL4 features seven plant homeodomain (PHD) fingers, which are methyl-lysine readers. These domains are organized as PHD1–3, PHD4–6, and a standalone PHD7, and may contribute to substrate recognition [17,24,25,26]. MLL4-mediated methylation proceeds via an ordered bi-bi mechanism, requiring prior binding of SAM to the active site [24].

Pathogenic mutations in MLL4 have been implicated in developmental syndromes (e.g., Kabuki syndrome [27], congenital heart disease [28]) and various cancers, including keratinocyte carcinomas, esophageal and pancreatic cancers, prostate cancer, and lymphomas [22,26,29,30]. Despite its critical roles in development and disease, H3K4 remains the only confirmed MLL4 target. This limitation highlights the need to identify additional, particularly non-histone, substrates. In this study, we employed a systematic in vitro and in silico approach to map the substrate landscape of MLL4, leading to the identification and characterization of CFP1 as a candidate non-histone substrate.

## 2. Methods

### 2.1. Protein Expression and Purification

Recombinant constructs for MLL4 (SET/Post-SET domain, residues 5382–5537), WDR5, RbBP5, Ash2L, Dpy-30, CFP1 (residues 302–656), and Setd1A (residues 1362–1563) were expressed in *Escherichia coli* BL21 cells. MLL4 was expressed as a GST fusion protein and purified by glutathione sepharose 4B affinity chromatography (GE Healthcare, Chicago, IL, USA). Cultures were grown in LB medium supplemented with 1 mM MgCl_2_ and 1 μM ZnSO_4_, induced with 0.1 mM IPTG at 16 °C for 18 h, then harvested by centrifugation.

GST-tagged MLL4 was purified following previously described techniques [31]. Bacterial lysis for MLL4 was performed using a GST lysis buffer (50mM Tris (pH 7.5), 300mM NaCl, 3mM dithiothreitol (DTT), 1 μM ZnSO_4_, 0.01% Triton X-100, 1mg/mL chicken egg lysozyme (Sigma, St. Louis, MO, USA), 10% glycerol) supplemented with a protease inhibitor cocktail containing 100 uM PMSF, 1 μM E-64, 1 μM pepstatin and 5 μM bestatin. For MLL4, lysis was followed by glutathione Sepharose 4B bead (GE Healthcare) affinity purification, and proteins were eluted using an elution buffer (50mM Tris (pH 8), 10mM glutathione).

All other proteins were expressed with 6 × His tags and purified using Ni-NTA agarose resin (Qiagen) following previously described techniques [32]. Bacterial lysis was performed using a P5 lysis buffer (50mM NaHPO_4_ (pH 7.0), 500mM NaCl, 3mM DTT, 0.05% Triton X-100, 1mg/mL chicken egg lysozyme (Sigma), 10% glycerol) supplemented with a protease inhibitor cocktail containing 100uM PMSF, 1 μM E-64, 1 μM pepstatin and 5 μM bestatin. The fusion proteins were then isolated from clarified lysate by Ni-NTA affinity chromatography and eluted using P500 (P5 with 500 mM imidazole).

Eluted proteins were dialyzed in a storage buffer (20mM Tris (pH 8), 100mM NaCl, 20% glycerol). The MLL4-WRAD complex was assembled by mixing equimolar purified MLL4, WDR5, RbBP5, Ash2L, and Dpy-30, quantified via Bradford Assay, and complex aliquots were snap-frozen in liquid nitrogen then stored at −80 °C.

### 2.2. SPOT Peptide Array Synthesis and Methylation Assay

Peptides were synthesized on aminated cellulose membranes using Fmoc chemistry on an Intavis Multipep synthesizer (0.2 μmol/spot). After deprotection (95% TFA, 3% TIPS, 2% H_2_O), arrays were washed with DCM, DMF, and ethanol, then dried and stored at room temperature. On array methylation followed previously described methods [33]. Arrays were rehydrated in ethanol and equilibrated in methylation buffer (50 mM Tris (pH 8.5), 3 mM MgCl_2_, 1 mM EDTA, 1 mM DTT). For methylation, arrays were incubated overnight at 4 °C with 2 μM MLL4-WRAD and 0.466 μM [^3^H]-SAM (PerkinElmer, Springfield, IL, USA) on a rocking platform. Following methylation, arrays were washed in a wash buffer (100 mM NH_4_HCO_3_, 1% sodium dodecyl sulfate (SDS)) followed by quick ethanol rinses. Arrays were saturated in 7% (*w*/*v*) 2,5-Diphenyloxazole (DPO) in ethanol, dried, wrapped, and exposed to autoradiographic film at −80 °C for 7 days. Developed films were analyzed densitometrically using ImageJ (v1.54h) (https://imagej.net/ij/index.html; accessed on 25 March 2024) with the Protein Array Analyzer macro as described previously [33].

### 2.3. In Vitro Methyltransferase Activity Assay

Activity was measured using the MTase-Glo™ kit (Promega, Madison, WI, USA, V7601) [34]. Dialyzed proteins were prepared in reaction buffer (20 mM Tris pH 8.0, 50 mM NaCl, 1 mM EDTA, 3 mM MgCl_2_, 1 mM DTT, 0.1 mg/mL BSA). Reactions included 15 μM substrate (either CFP1 or H3K4), 10 μM SAM, and 0.5 μM assembled MLL4-WRAD complex. Reactions (8 μL total) were incubated (n = 4) in 384-well plates for 16 h at room temperature in the dark. Following incubation, 2 μL of 5× MTase-Glo reagent was added and incubated for 1 h, followed by 10 μL detection solution and a further 1 h incubation. Luminescence was measured using a BioTek Cytation 5 plate reader. Data were analyzed using one-way ANOVA in GraphPad Prism 7.

### 2.4. Peptide Binding Array with MLL4 PHD Domains

Arrays were rehydrated and equilibrated with 0.1% TBS-T, then incubated overnight at 4 °C with 1 μM of MLL4 PHD1–3 (residues 156–341), PHD4–6 (1358–1572), or PHD7 (5079–5152) in TBS-T. After washing, arrays were blocked with 0.5% non-fat milk in TBS-T for 1 h and probed with anti-6 × His-HRP in TBS-T (1:5000) for 2 h. Chemiluminescence was developed with Bio-Rad Clarity ECL and imaged using a ChemiDoc XRS+ system. Signal intensities were normalized to the H4K20me0 peptide, which served as an internal reference as it is a known ligand of PHD4–6 [35]. For each array, the intensity of H4K20me0 was set to 100%, and all other peptide signals were expressed relative to this value. Spots that reached at least 50% of the normalized H4K20me0 signal were considered candidate binders.

### 2.5. Cloning of Setd1A Fragment

Setd1A (residues 1362–1563) was PCR-amplified using primers (F: 5′-TAAGCAAAGCTTGCGAGGAGGGCGAAGAGGAG-3′; R: 5′-TAAGCACTCGAGGCCCATGATGGCGGAGGTACC-3′). The amplicon and pET28a vector were digested with HindIII and XhoI and ligated (1:10 ratio) using T4 DNA ligase. Ligation products were transformed into DH5α and BL21 cells. Positive clones were confirmed by colony PCR and sequencing.

### 2.6. CFP1 Methylation and Pulldown Assay

Recombinant CFP1 (302–656) was cleaved with Thrombin CleanCleave (Sigma) and dialyzed into methylation buffer. CFP1 (5 μM) was incubated overnight at room temperature with 0.2 μM MLL4-WRAD and 10 μM SAM. Glutathione Sepharose 4B beads were added to bind the MLL4 complex. After 2 h incubation, beads were washed with increasing NaCl (Δ0 to Δ500 mM) to remove nonspecific interactions. Eluted CFP1me (302–656) was concentrated by speed-vac to near dryness, and reconstituted in PBS for subsequent binding study. 0.25 μM of CFP1 or CFP1me was incubated with 0.75 μM Setd1A (1362–1563) in PBS overnight at 4 °C. The reaction was incubated with Ni-NTA beads, and wash fractions were collected at increasing NaCl concentrations. Samples were analyzed by SDS-PAGE and Coomassie staining using a Bio-Rad ChemiDoc system (Bio-Rad, Hercules, CA, USA).

## 3. Results

### 3.1. A Systematic Approach Uncovers CFP1 K328 as an MLL4 Substrate Candidate

**Figure 1:**
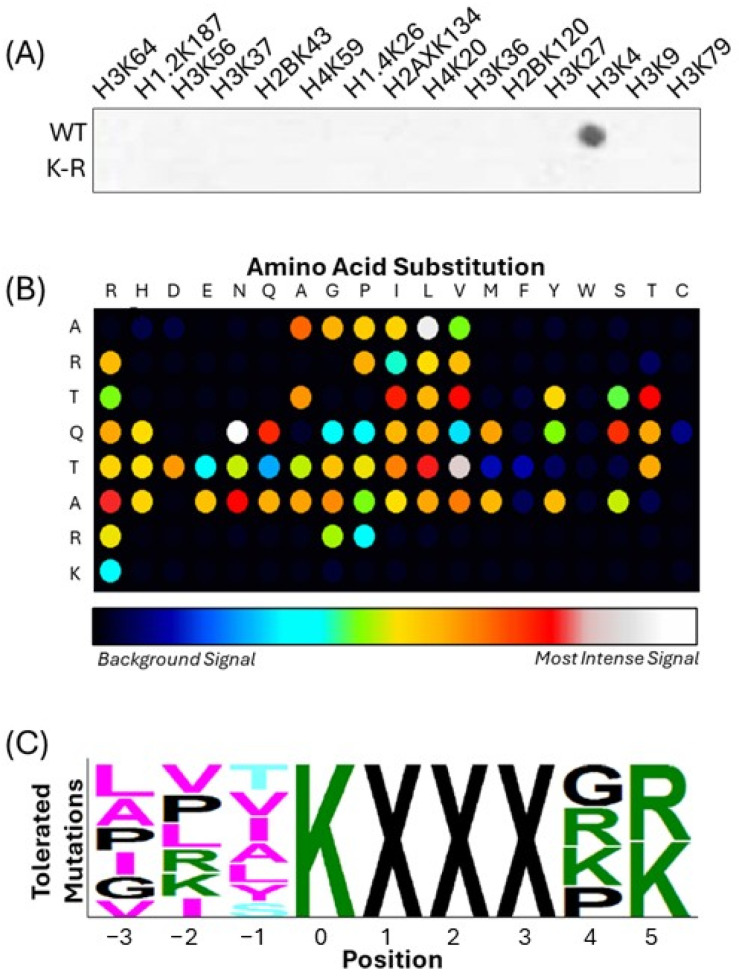
Identification of the MLL4 substrate recognition motif using SPOT peptide arrays. (**A**) A control array of histone tail peptides demonstrates that the MLL4-WRAD complex exhibits methyltransferase activity predominantly on H3K4, confirming substrate specificity. Peptides with K-to-R substitutions (bottom row) serve as negative controls. Dark spots indicate incorporation of radiolabeled [^3^H]-SAM, signifying methyl group transfer. (**B**) A permutation array of the H3 (1–9) peptide was used to assess the contribution of each residue to MLL4 recognition and H3K4 peptide methylation. Each wild-type residue was systematically replaced with all 20 amino acids, and signal intensity indicates methylation efficiency. (**C**) A sequence motif was generated using PeSA 2.0 software [36], summarizing the tolerated and preferred residues at each position relative to the methylated lysine (position 0).

To systematically uncover MLL4 substrates, we first began by screening arrayed peptide representatives of known histone methylation sites with a recombinant MLL4-WRAD complex, using K-to-R mutant peptides as negative controls (Figure 1A). MLL4 preferentially methylated the H3K4 representative peptide, while methylation was abolished in the K-to-R mutant, confirming substrate specificity. This validated the peptide ARTKQTARK as a bona fide in vitro target of MLL4-WRAD and established it as a positive control for subsequent experiments. To define substrate preferences, we then synthesized a permutation array of the H3K4 methylation window, comprising three N-terminal and five C-terminal flanking residues surrounding the modified lysine (e.g., ART[K]QTARK), and assessed methylation with [^3^H]-SAM and MLL4-WRAD. In designing the permutation array, each position within the defined flanking region was systematically substituted with all 20 canonical amino acids, while the lysine acceptor site was held constant. This approach generates a comprehensive library of sequence variants in which the influence of every possible residue at each position can be independently evaluated. By comparing the relative methylation signals across the array, we were able to derive positional preferences of MLL4, identifying both favored and disfavored residues adjacent to the methyl-acceptor lysine. Signal quantification and modeling (Figure 1B) enabled generation of a consensus substrate motif using PeSA (Figure 1C). Using a 50% threshold of H3K4 methylation signal, we defined MLL4’s substrate motif as: [LAPIGV]-[VPLRKI]-[TVIALYS]-K-[X]-[X]-[X]-[GRKP]-[RK], whereby ‘X’ denotes permissive positions.

**Figure 2:**
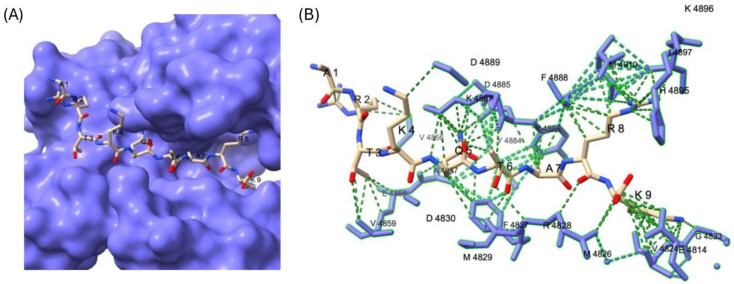
Predicted interaction of the H3K4 peptide with the MLL3 SET domain binding pocket. (**A**) Surface representation of the MLL3 SET domain (residues 4757–4910) with the docked H3K4 peptide (ARTKQTARK) shown in stick format. (**B**) Detailed view of peptide–protein interactions within 5 Å, highlighting contacts between key H3K4 residues and residues in the MLL3 substrate binding pocket. The methyl-accepting lysine (K4) is positioned in the catalytic channel, flanked by extensive interactions with side chains lining the groove. Docking was performed using HPEPDOCK, and structural visualization was carried out using ChimeraX. MLL3 SET domain coordinates were obtained from the crystal structure described in [17] (PDB: 5F59).

To explore potential structural determinants, we modeled MLL4-H3K4 peptide binding using the available MLL3 crystal structure (PDB 5F59), due to conserved binding residues across the MLL family [17,37]. Docking analysis (Figure 2) revealed limited contacts between the +1, +2, and +3 residues and the binding pocket—explaining their tolerance for substitution—while c-terminal positions with tighter pocket interaction aligned with residues exhibiting low substitution tolerance.

Next, we then queried a nuclear protein lysine database (218,254 residues) using the 9-residue motif. This returned 452 perfect motif matches. Only 192 of which were within proteins previously identified as methylated (Supplemental Table S1), while just five proteins had reported methylation at the lysines identified from our motif (Table 1); this includes CFP1 (CXXC finger protein 1), a Setd1A/B-associated factor, which was selected for further analysis based on biological relevance. Within CFP1, K328 emerged as a candidate methylation site. This residue had previously been identified in proteome screens as methylated [38] and lies within the basic region of CFP1, just upstream of the Setd1-interaction domain (SID). Notably, the SID is critical for mediating CFP1-Setd1 association and downstream Setd1 methyltransferase activity [39]. Given that point mutations in the SID abrogate Setd1 binding [39], potential modification of this proximal region is of particular interest. A parallel, shotgun-style substrate discovery approach also yielded positive array results (Supplemental Figure S1), though methylation was not confirmed in vitro.

### 3.2. CFP1 Can Undergo In Vitro Methylation Mediated by MLL4/WRAD at Multiple Sites

**Figure 3:**
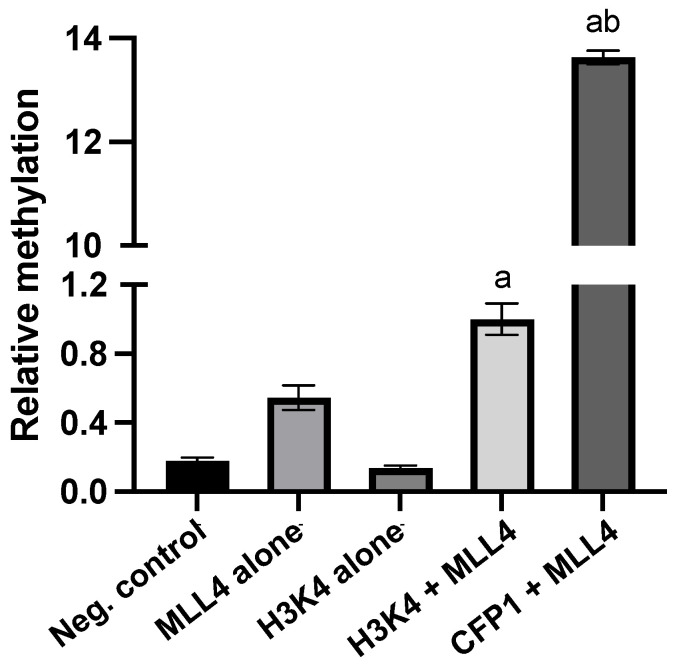
MLL4-WRAD complex methylates recombinant CFP1(302–656) in a bioluminescence-based methyltransferase assay. Methylation activity was assessed using the MTase-Glo™ assay (Promega, Cat# V7601), which measures S-adenosylhomocysteine (SAH) production as a luminescent readout. Reactions were performed at room temperature for 16 h in the dark and processed according to the manufacturer’s protocol. Luminescence was measured using a BioTek Cytation 5 plate reader. Bars represent mean ± SD (n = 4). ‘a’ indicates a significant difference from MLL4 alone; ‘b’ indicates a significant difference from the H3K4 + MLL4 condition (*p* < 0.05).

**Figure 4:**
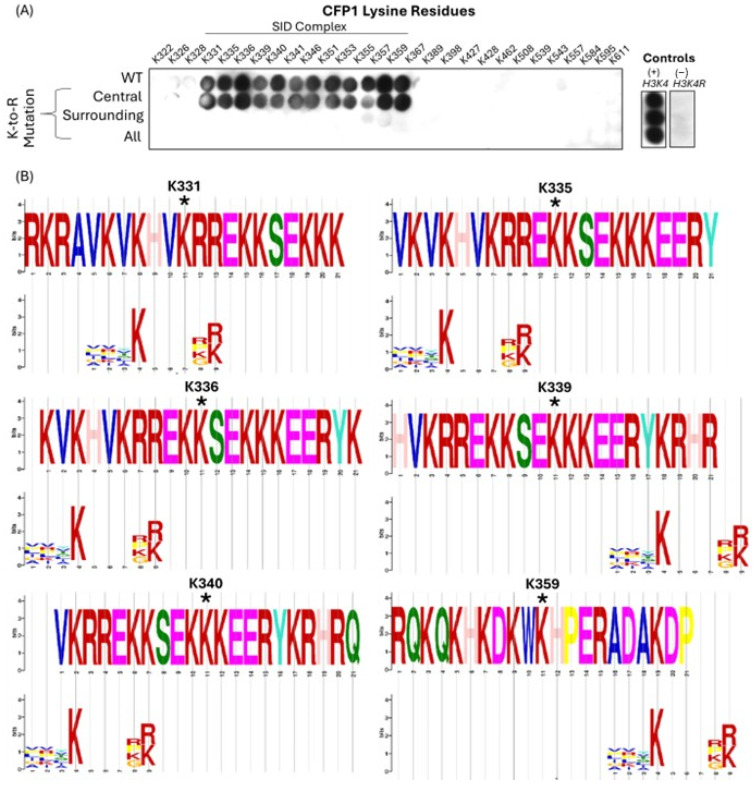
Identification of putative methylation sites on CFP1 by MLL4-WRAD complex. (**A**) Incorporation of radiolabeled [^3^H]-SAM was measured using an on-array methylation assay, assessing lysine-containing peptides spanning all lysine residues within the CFP1(302–656) construct. Peptides were synthesized as wild-type (WT), with central lysine-to-arginine (K→R) substitutions, surrounding residue substitutions, or all lysine residues mutated. Signal intensity indicates methylation activity. H3K4 and H3K4R peptides were used as positive and negative controls, respectively. (**B**) Sequence logos comparing local CFP1 peptide sequences surrounding individual lysine sites (**top**, centered on the methylated lysine) to the derived MLL4 substrate motif (**bottom**). Generated using Tomtom [43]. Asterisks denote the central lysine position in each CFP1 sequence.

Using the MTase-Glo™ luminescence-based methyltransferase activity assay (Promega), we observed MLL4/WRAD-mediated methylation of CFP1 at multiple sites (Figure 3). To investigate further, we generated a peptide array covering all lysines in CFP1 (302–656) and probed for methylation with recombinant MLL4/WRAD and [^3^H]-SAM (Figure 4A). Multiple CFP1 peptides were methylated: K331, K335, K336, K339, K340, K341, K346, K351, K353, K355, K357, and K359. Surprisingly, K328 (initially identified via motif search due to its known methylation status) showed minimal signal compared to these other sites. We next assessed motif alignment by extracting 21-residue “methylation windows” (10 residues flanking each side of the central lysine) and comparing them to the motif in Figure 1C. Six methylation sites (K331, K335, K336, K339, K340, and K359) showed alignment (Figure 4B). Notably, windows surrounding K331 and K335 include K328, suggesting K328’s inclusion may contribute to MLL4 targeting. These methylation sites fall within the basic region of CFP1 (residues 321–360), a domain initially suggested to mediate homodimerization based on low-mobility EMSA shifts for CFP1 fragments containing this region [40]. At the time, the SID had not yet been discovered, and the fragment studied (residues 106–345) spanned both the basic domain and part of the SID. More recently, CFP1 has been shown to form homodimers in yeast [41], and quantitative proteomics revealed that CFP1 is 1.6× more abundant than the Setd1A/B catalytic subunit in pull-down experiments [42]. Although poorly studied, the basic domain may influence Setd1 binding. Butler et al. [39] demonstrated that deleting the basic region (residues 302–361) from CFP1 reduced Setd1A binding by ~50%. Together, these findings support a hypothesis that MLL4-mediated methylation in the basic region could modulate CFP1 interactions and potentially regulate dimerization.

### 3.3. MLL4/WRAD-Mediated CFP1 Methylation Affects MLL4 PHD Cassette Binding

**Figure 5:**
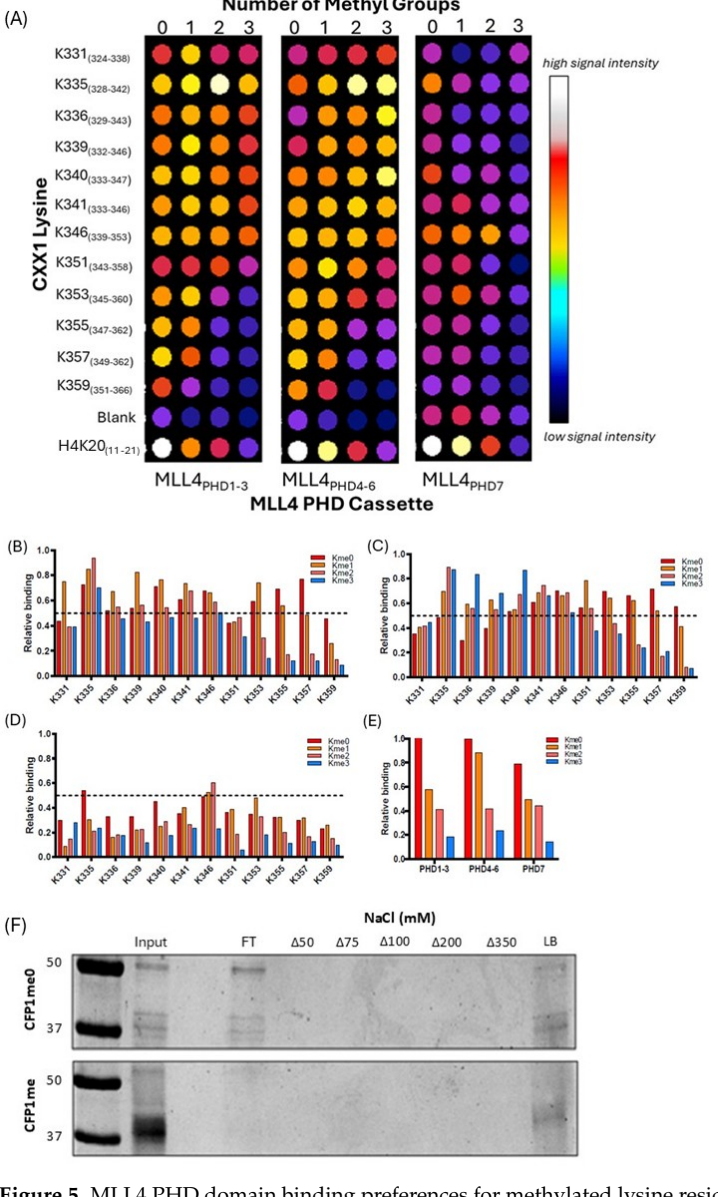
MLL4 PHD domain binding preferences for methylated lysine residues in the CFP1 CXXC1 SID region. (**A**) Dot blot array showing binding of three MLL4 PHD cassettes (PHD1–3, PHD4–6, and PHD7) to peptides representing lysine-centered methylation windows within the CFP1 SID region. Each peptide was synthesized with lysine in one of four methylation states: unmodified (Kme0), mono- (Kme1), di- (Kme2), or trimethylated (Kme3). Binding intensity was visualized using ImageJ and the intensity of H4K20me0 was set to 100%, and all other peptide signals were expressed relative to this value. Spots that reached at least 50% of the normalized H4K20me0 signal were considered candidate binders. (**B**–**D**) Quantification of binding affinity for each PHD cassette to the indicated CFP1 lysine peptides across methylation states. (**B**) PHD1–3, (**C**) PHD4–6, and (**D**) PHD7. (**E**) Summary plot comparing relative methylation preference of each MLL4 PHD cassette across all tested sites. (**F**) Pulldown assay demonstrating selective binding of methylated CFP1 to the Setd1A SID. Recombinant CFP1 (302–656) methylated by MLL4-WRAD (**top panel**) or unmethylated (**bottom panel**) was incubated with 6xHis-tagged Setd1A (1362–1563) immobilized on Ni-NTA resin.

To explore how site-specific lysine methylation affects CFP1’s interactions with MLL4, we synthesized arrays of peptides centered on each identified methylation site (±10 flanking residues), in unmethylated, mono-, di-, and tri-methylated forms. These were probed with bacterially expressed recombinant MLL4 PHD1–3, PHD4–6, and PHD7 domain constructs. Binding was clearly influenced by methylation state and residue identity (Figure 5A).

Specifically, PHD1–3 preferentially bound K331me1 and K339me1, with significantly weaker interaction at me0, me2, or me3 states (Figure 5B). K335 bound PHD1–3 across all methylation states, with strongest relative binding observed for K335me3. In contrast, SID-proximal residues (K355, K357, K359) showed binding only in me0 and me1 states, with me2 and me3 abolishing interaction, suggesting a potential role for hydrogen bonding and structural recognition. These three residues are conserved among mammals, highlighting their possible biological importance. A similar methylation-state dependency was seen with PHD4–6 (Figure 5C). K335 again bound in all methylation states, but affinity increased with me2 and me3. K335me3, K340me3, and K351me2 showed strongest binding. Like PHD1–3, PHD4–6 binding to SID-proximal residues was lost at higher methylation states. PHD7, in contrast, bound strongly to unmethylated K335 and showed reduced interaction with K345me3 (Figure 5D). Although PHD domains are best known for combinatorial reading of histone PTMs [1,3], these results suggest that MLL4 PHD domains may also engage non-histone methyl marks in a residue- and methylation state-specific manner. This opens the door for further exploration of their role in non-histone epigenetic regulation. All domain interactions were quantified relative to H3K4me0 peptide binding.

### 3.4. MLL4/WRAD-Mediated CFP1 Methylation Modulates Setd1A Interaction

To evaluate the functional relevance of CFP1 methylation, we tested whether it influenced Setd1A binding in vitro. In pulldown assays, methylated CFP1me (302–656) co-eluted only upon bead denaturation (LB), suggesting strong interaction with Ni-NTA-bound Setd1A (1362–1563). Bound proteins were eluted under increasing NaCl concentrations (Δ50–Δ350 mM) or boiled in Laemmli buffer (LB). Input, flowthrough (FT), and elution fractions are shown. Only methylated CFP1 binds Setd1A, as indicated by elution in LB. In contrast, unmethylated CFP1 was detected in the flow-through (FT), indicating a lack of association. These results (Figure 5F) support a model in which MLL4-mediated methylation enables or stabilizes CFP1–Setd1A binding—a potential methyl-switch mechanism. This interaction may have broader implications. MLL4, Setd1A, and CFP1 are known to localize to distinct nuclear compartments: MLL4 is enriched at distal enhancers; Setd1A at actively transcribed promoters [15,44]; and unbound CFP1 in nuclear speckles—dynamic bodies associated with gene expression [45]. It is plausible that MLL4 methylates CFP1 in speckles, enabling its translocation to Setd1A complexes, or that enhancer regions are spatially proximal to transcription sites, allowing tri-protein interaction. Taken together, our findings support a potential model in which MLL4 indirectly regulates H3K4 di- and tri-methylation via CFP1 methylation and its subsequent association with Setd1A/B. This proposed axis of regulation warrants deeper investigation.

## 4. Discussion

MLL4 is a well-established H3K4 lysine methyltransferase (KMT) that plays essential roles in development, transcriptional regulation, and enhancer function [23,24]. It is a core member of the COMPASS complex and contributes to Setd1 activity [7,17]. Although MLL4 mutation and dysregulation have been implicated in several disease states [26,27,28,29,30], H3K4 remains its only canonical substrate. In this study, we investigated whether MLL4 targets non-histone proteins, identifying CFP1 as a substrate candidate using a combination of rational motif-based screening, in vitro methylation assays, and interaction studies.

We first derived a substrate preference motif for MLL4 through permutation screening of the H3K4 methylation window. Using this motif, we queried nuclear proteins and identified five lysine residues with perfect alignment: TOP2A K1286, histone 3.1 K5, KAT2B K672, TSPYL2 K407, and CFP1 K328 (Table 1). While systematic approaches such as this help minimize researcher bias compared to “shotgun” strategies, they also inherently favor sequences similar to known substrates and do not account for biological context. Importantly, CFP1 was selected for downstream validation due to its known roles as a key member of the Setd1A/B COMPASS complexes [39,45] and as a binding partner of the DNA CpG methyltransferase DNMT1 [39,41].

Using a bioluminescent methyltransferase assay (Promega MTase-Glo™), we showed that MLL4/WRAD methylates a peptide representative of CFP1 K328 with higher activity than the canonical H3K4 substrate (Figure 3). However, SPOT array screening of all lysines in CFP1 (302–656) revealed twelve methylated residues—K331, K335, K336, K339, K340, K341, K346, K351, K353, K355, K357, and K359—with K328 notably absent (Figure 4A). These residues cluster within the basic region of CFP1, a domain previously implicated in protein interaction and homodimerization [39,40]. Notably, K331 first appeared in vertebrates, while K355, K357, and K359 are mammal-specific (Supplemental Figure S1), raising the possibility that these evolved in tandem with MLL4’s regulatory functions. However, this evolutionary hypothesis would require substantial additional validation. We were curious whether the observed methylation sites aligned with the motif generated in Figure 1C. Although the motif was successful in predicting K328, none of the twelve experimentally methylated lysines aligned with it. In fact, only windows that included K328 showed perfect motif alignment (Figure 4B). This underscores the limitation of motif-based predictions: while useful as a rational starting point, they cannot fully account for structural or contextual factors that govern enzyme–substrate interactions.

To better understand MLL4’s recognition of these methylation events, we screened each of the twelve methylated CFP1 peptides across all methylation states (me0–me3) for binding to the three PHD cassettes of MLL4 (Figure 5A). PHD domains are traditionally seen as histone mark readers [1,3], but emerging work has shown that they can engage non-histone methylation targets [46,47]. We observed residue- and methylation-specific binding interactions—for example, PHD1–3 preferentially bound K331me1 and K339me1, while PHD4–6 showed increased interaction with K335me3 and K351me2 (Figure 5B–D). MLL4 PHD4–6 has previously been shown to bind non-histone targets such as TET3 [48], suggesting these interactions may have broader biological significance.

We then examined the effect of CFP1 methylation by MLL4/WRAD on its interaction with Setd1A using pulldown assays (Figure 5F). Methylated CFP1 bound Setd1A, whereas unmethylated CFP1 did not, suggesting a switch-like mechanism of regulation. This finding positions MLL4/WRAD as a possible upstream regulator of Setd1A/B activity via CFP1 methylation. Although MLL4, Setd1A, and CFP1 are known to localize to distinct nuclear compartments—distal enhancers, active promoters, and nuclear speckles, respectively [15,43,49]—our results raise the possibility that MLL4-mediated methylation of CFP1 facilitates its transition into Setd1-containing complexes. Whether this occurs through spatial proximity at enhancer–promoter loops or via nuclear speckle-associated exchange remains to be determined.

Together, these findings support a candidate model in which CFP1 is a non-histone substrate of MLL4 and that methylation within its basic region may modulate Setd1A/B complex assembly. Additional work is needed to validate these interactions in a cellular context, as the present study explores the CFP1/MLL4/WRAD interaction in vitro exclusively. Exploring this interaction in cells will provide greater evidence of both the global levels of methylated CFP1 as a function of MLL4 expression, as well as demonstrating the functional implications of the methylation event. Regardless, this study expands the substrate landscape of MLL4 and suggests a regulatory role in COMPASS function that extends beyond histone modification.

## 5. Conclusions

This work provides preliminary in vitro evidence implicating CFP1 as a non-histone methylation target of MLL4 and establishes a foundation for further investigation into MLL4’s extended substrate network. The identification of CFP1 methylation sites within the lysine-rich basic domain and the observed switch-like regulation of Setd1A interaction suggest that MLL4 exerts broader influence over chromatin-modifying complexes than previously appreciated. Future studies examining these interactions in vivo and within chromatin contexts will be essential to defining the full scope of MLL4’s biological roles.

## Figures and Tables

**Table 1:** MLL4/WRAD motif perfect match sequences with known methyl lysine modified residues.

Uniprot ID	Gene Name	Residue	Sequence
P11388	TOP2A_HUMAN	1286	AFKPIKKGKKR
P68431	H31_HUMAN	4	ARTKQTARK
Q92831	KAT2B_HUMAN	672	KEIIKKLIERK
Q9H2G4	TSPYL2_HUMAN	407	RIKRKKQEMKK
Q9P0U4	CXXC1_HUMAN	328	RAVKVKHVKRR

## Data Availability

Dataset available on request from the authors.

## Supplementary Materials

The following supporting information can be downloaded at: https://www.mdpi.com/article/10.3390/epigenomes9040041/s1, Table S1: MLL4 WRAD motif perfect match sequences in known methyllysine proteome; Figure S1: (A) Cladogram illustrating the evolutionary relatedness of CFP1 in *H. sapiens* (Human), *M. Musculus* (Mouse) *A. carolinensis* (Lizard) *D. rerio* (Fish) *D. melanogaster* (Fly) *C. elegans* (Worm), and *S. cerevisiae* (Yeast). (B) Methylated lysines in the basic region of CFP1 are conserved.
