# Supplementary material for: Exploration into the MLL4/WRAD Enzyme-Substrate Network: Systematic In Vitro Identification of CFP1 as a Potential Non-Histone Substrate of the MLL4 Lysine Methyltransferase
Source: Epigenomes. 2025 Oct 15;9(4):41. doi: 10.3390/epigenomes9040041 (PMC12551112; doi:10.3390/epigenomes9040041)
Supplement: Supplementary file 1 [file epigenomes-09-00041-s001.zip › epigenomes-3739750-supplementary.pdf]

Supplemental Table S1. MLL4 WRAD motif perfect match sequences in known methyllysine proteome.

Uniprot ID	Gene Name	Residue	Sequence
O00567	NOP56_HUMAN	448	EKKRLKKEKKR
O14646	CHD1_HUMAN	1498	LHKLYKHAIKK
O15391	TYY2_HUMAN	143	SKKPSKKPSGK
O43290	SNUT1_HUMAN	403	TFKKTKRRVKK
O43395	PRPF3_HUMAN	110	SSGVKKRRIPR
O60673	REV3L_HUMAN	1189	PSIVTKKRNKR
O60885	BRD4_HUMAN	285	QPVKTKKGVKR
O75151	PHF2_HUMAN	696	FPIRRKKNAPK
O75151	PHF2_HUMAN	953	KSKKKKSAKRK
O75367	H2AY_HUMAN	147	KKPVSKKAGGK
O75376	NCOR1_HUMAN	2329	PKLISKSNSRK
O75528	TADA3_HUMAN	124	GRPKSKNLQPK
O75592	MYCB2_HUMAN	2823	APVKTKLDPPR
O75592	MYCB2_HUMAN	3168	ENKKSKKEKKK
O76021	RL1D1_HUMAN	347	EHGKKKRGRGK
O95260	ATE1_HUMAN	247	FPPKAKSNQPK
O95359	TACC2_HUMAN	2287	NPPPTKKIGKK
O96028	NSD2_HUMAN	1080	WGLVAKRDIRK
P00533	EGFR_HUMAN	676	RHIVRKRTLRR
P07305	H10_HUMAN	111	AFKKTKKEIKK
P07900	HS90A_HUMAN	414	LKVIRKNLVKK
P08238	HS90B_HUMAN	406	LKVIRKNIVKK
P10242	MYB_HUMAN	120	WSVIAKHLKGR
P11388	TOP2A_HUMAN	1461	DPAKTKNRRKR
P11388	TOP2A_HUMAN	1286	AFKPIKKGKKR
P16402	H13_HUMAN	179	SAKKVKTPQPK
P16403	H12_HUMAN	22	KAPVKKKAAKK
P25490	TYY1_HUMAN	173	GGGRVKKGGGK
P27695	APEX1_HUMAN	27	EAKKSKTAAKK
P31629	ZEP2_HUMAN	2280	PYVLSKQHEKR
P38432	COIL_HUMAN	183	EEAKRKSPKKK
P43243	MATR3_HUMAN	478	KYKRIKKPEGK
P46013	KI67_HUMAN	2647	GIKVLKQRAKK
P46087	NOP2_HUMAN	699	VTGKLKQRSPK
P48382	RFX5_HUMAN	465	SDAKRKRGRPR
P48634	PRC2A_HUMAN	1190	WSPPAKSLAPK
P49711	CTCF_HUMAN	259	PTKIKKKGVKK
P49711	CTCF_HUMAN	595	ETKKSKRGRKR
P49711	CTCF_HUMAN	649	APPPAKKRRGR
P49756	RBM25_HUMAN	236	SHPRKKKKEKK
P49792	RBP2_HUMAN	614	VLPLLKIIKKK
P51532	SMCA4_HUMAN	583	KEKKKKKKKKK
P51532	SMCA4_HUMAN	1596	RSVKVKIKLGR
P55769	NH2L1_HUMAN	86	VFVRSKQALGR
P68431	H31_HUMAN	5	AMARTKQTARK
P78527	PRKDC_HUMAN	2908	AELPAKRVRGK

P78545	ELF3_HUMAN	246	KHGKRKRGRPR
Q03164	KMT2A_HUMAN	234	PGVKIKITHGK
Q03164	KMT2A_HUMAN	272	ATKIKKLRAGK
Q03164	KMT2A_HUMAN	959	TAVKTKILIKK
Q05639	EF1A2_HUMAN	439	AVGVIKNVEKK
Q09666	AHNK_HUMAN	5711	KLKKSKIKMPK
Q12873	CHD3_HUMAN	283	PDGRKKLRGKK
Q12873	CHD3_HUMAN	51	GVKKRKRGPKK
Q12906	ILF3_HUMAN	388	PSKKKKKIQKK
Q13023	AKAP6_HUMAN	600	VPLLSKHKSKK
Q13111	CAF1A_HUMAN	795	AAIPSKSRLKR
Q13123	RED_HUMAN	300	NKKLKKKDKGK
Q13330	MTA1_HUMAN	527	SPLVLKQAVRK
Q13415	ORC1_HUMAN	460	TKVPKKSLKPR
Q13428	TCOF_HUMAN	1439	SNPKSKKEKKK
Q13435	SF3B2_HUMAN	451	APKLSKKKLRR
Q13523	PRP4B_HUMAN	224	ERKKSKSPSKR
Q13620	CUL4B_HUMAN	795	ARVLAKNPKGK
Q13823	NOG2_HUMAN	654	DRAPSKKGKKR
Q13895	BYST_HUMAN	194	REVLSKYRSGK
Q14498	RBM39_HUMAN	51	ERKRSKSKERK
Q14498	RBM39_HUMAN	67	ERKKSKSRERK
Q14562	DHX8_HUMAN	703	FGLLKKTVQKR
Q14669	TRIPC_HUMAN	1003	DVLKRKRLPKR
Q14781	CBX2_HUMAN	162	KDPIRKKRGRK
Q14865	ARI5B_HUMAN	445	GTKRIKHEIPK
Q14978	NOLC1_HUMAN	76	NGPVAKKAKKK
Q14978	NOLC1_HUMAN	615	TFPKRKKGEKR
Q15046	SYK_HUMAN	20	EPKLSKNELKR
Q15059	BRD3_HUMAN	239	PPVVKKKGVKR
Q15059	BRD3_HUMAN	490	VNKPKKKKEKK
Q15361	TTF1_HUMAN	276	HKKKSKKKKKK
Q15398	DLGP5_HUMAN	22	EMIRTKIAHRK
Q15545	TAF7_HUMAN	141	ITLPLKNVRKR
Q1ED39	KNOP1_HUMAN	65	EMPLVKKKKKK
Q1ED39	KNOP1_HUMAN	66	MPLVKKKKKKK
Q1ED39	KNOP1_HUMAN	67	PLVKKKKKKKK
Q1ED39	KNOP1_HUMAN	289	PALKRKKKKKR
Q1ED39	KNOP1_HUMAN	290	ALKRKKKKKRK
Q32MZ4	LRRF1_HUMAN	570	SQKKTKNKKKK
Q32MZ4	LRRF1_HUMAN	575	KNKKKKNKKKK
Q3L8U1	CHD9_HUMAN	2429	NQPIVKKRRGR
Q3L8U1	CHD9_HUMAN	2430	QPIVKKRRGRR
Q562F6	SGO2_HUMAN	614	INKLRKKVNRK
Q56NI9	ESCO2_HUMAN	547	RLKRRKRIARR
Q5T8A7	PPR26_HUMAN	704	DLLRSKRKLKK
Q66PJ3	AR6P4_HUMAN	297	KDKRRKKKKKR
Q66PJ3	AR6P4_HUMAN	303	KKKKRKKLKKK
Q6PD62	CTR9_HUMAN	933	DLPISKKKKRR
Q6PD62	CTR9_HUMAN	983	GPKPKKRRPPK
Q6UB98	ANR12_HUMAN	1845	LHIRKKIEEKR

Q6UB99	ANR11_HUMAN	2447	LQIRKKIEEKR
Q7KZF4	SND1_HUMAN	409	EFLRKKLIGKK
Q7Z699	SPRE1_HUMAN	322	SLKIKKSKRRK
Q7Z6E9	RBBP6_HUMAN	1731	SKKKKKKKEKK
Q7Z6E9	RBBP6_HUMAN	1732	KKKKKKKEKKK
Q86UE4	LYRIC_HUMAN	443	PTGKSKKKKKK
Q86UE4	LYRIC_HUMAN	446	KSKKKKKKKKK
Q86UE4	LYRIC_HUMAN	575	KQIKKKKKARR
Q86X95	CIR1_HUMAN	237	EKKKKKKDRKK
Q86XP3	DDX42_HUMAN	298	MIGIAKTGSGK
Q8IVF2	AHNK2_HUMAN	3614	QAPKAKLDAGR
Q8IVL0	NAV3_HUMAN	2076	EYVITKSGRKK
Q8IWS0	PHF6_HUMAN	159	SSPKSKKKSRK
Q8IY81	SPB1_HUMAN	804	TYVVAKKGVGR
Q8IY92	SLX4_HUMAN	1661	PKGPAKTKGPR
Q8IZA3	H1FOO_HUMAN	174	NVGKVKKAAKR
Q8IZT6	ASPM_HUMAN	674	FIKPLKTDIPR
Q8N8L2	ZN491_HUMAN	76	LEKPYKHKQRR
Q8NEZ4	KMT2C_HUMAN	3605	EKGKKKRTRKK
Q8NF91	SYNE1_HUMAN	6675	ASAVLKRAHKR
Q8NI36	WDR36_HUMAN	773	LDVIKKKNKPK
Q8TDD1	DDX54_HUMAN	752	DKKKIKTESGR
Q8TDI0	CHD5_HUMAN	50	VSLPKKKKPKK
Q8TDI0	CHD5_HUMAN	324	LGKKSKRRRKK
Q8TEK3	DOT1L_HUMAN	397	SKARKKKLNKK
Q8TEQ6	GEMI5_HUMAN	828	KVILLKKEPPK
Q8WUI4	HDAC7_HUMAN	167	RYKPKKSLERR
Q8WYP5	ELYS_HUMAN	1973	SAIPRKRGRPR
Q8WZ42	TITIN_HUMAN	2516	DEGPYKLIVGR
Q8WZ42	TITIN_HUMAN	10606	EEKVLKLKPKR
Q8WZ42	TITIN_HUMAN	11877	EEVVLKSVLRK
Q8WZ42	TITIN_HUMAN	17902	TCKVSKLLEGK
Q8WZ42	TITIN_HUMAN	31951	VEVPAKIHLPK
Q8WZ42	TITIN_HUMAN	33594	SPPRVKSPEPR
Q92541	RTF1_HUMAN	229	IKKKLKTAKKK
Q92831	KAT2B_HUMAN	672	KEIIKKLIERK
Q96BK5	PINX1_HUMAN	295	TLKPKKRRGKK
Q96BR9	ZBT8A_HUMAN	199	QQPLAKHEPRK
Q96JP5	ZFP91_HUMAN	286	EEPPRKRGRRR
Q96KM6	Z512B_HUMAN	859	EEPVAKLPPRR
Q96KQ7	EHMT2_HUMAN	361	SRKRRKREPPR
Q96L73	NSD1_HUMAN	1959	WGLRTKTDIKK
Q96L91	EP400_HUMAN	1432	AELLSKKKIPR
Q96Q15	SMG1_HUMAN	2134	TILPTKTKPKK
Q96QE3	ATAD5_HUMAN	479	KNKKLKKKNKK
Q9BQ70	TCF25_HUMAN	131	RKKKKKQKNKK
Q9BQG0	MBB1A_HUMAN	1167	QSPISKKRKKK
Q9BQI6	SLF1_HUMAN	313	YTLRRKRKKGK
Q9BVI0	PHF20_HUMAN	543	RVKPKKKKKKK
Q9BVI0	PHF20_HUMAN	544	VKPKKKKKKKK
Q9BVI0	PHF20_HUMAN	545	KPKKKKKKKKK

Q9BVP2	GNL3_HUMAN	7	KRPKLKKASKR
Q9BW19	KIFC1_HUMAN	89	AQKVSKKTGPR
Q9BYG3	MK67I_HUMAN	174	ERLLRKKLAKK
Q9BZ95	NSD3_HUMAN	1306	KNAKLKQKRRK
Q9H0H5	RGAP1_HUMAN	179	TFKLKKREKRR
Q9H2G4	TSYL2_HUMAN	407	RIKRKKQEMKK
Q9H2P0	ADNP_HUMAN	616	AAVPYKKDVGK
Q9H2Y7	ZN106_HUMAN	1040	KNKRRKIKGKK
Q9H2Y7	ZN106_HUMAN	1353	GSKKKKKLRKK
Q9H2Y7	ZN106_HUMAN	1354	SKKKKKLRKKK
Q9H6F5	CCD86_HUMAN	329	NPAKLKRAKKK
Q9HCG8	CWC22_HUMAN	822	GDPKKKRGERR
Q9NPF5	DMAP1_HUMAN	292	KAPKKKLPQKK
Q9NPF5	DMAP1_HUMAN	86	RTVKAKLGSKK
Q9NPG3	UBN1_HUMAN	186	KEKKKKSPKKR
Q9NQZ2	SAS10_HUMAN	342	LIPKAKSTKPK
Q9NR30	DDX21_HUMAN	75	NSPKSKKAKKK
Q9NR30	DDX21_HUMAN	56	VFPKAKQVKKK
Q9NR30	DDX21_HUMAN	93	ISPKTKSLRKK
Q9NR48	ASH1L_HUMAN	711	SKPLKKRKGRK
Q9NR48	ASH1L_HUMAN	710	QSKPLKKRKGR
Q9NRL2	BAZ1A_HUMAN	1357	LSPRRKRRGRK
Q9NUQ6	SPS2L_HUMAN	66	MTGKKKNNKRK
Q9NVU7	SDA1_HUMAN	272	MKVLKKQKKKK
Q9NZI8	IF2B1_HUMAN	77	HSVPKKQRSRK
Q9P0U4	CXXC1_HUMAN	328	RAVKVKHVKRR
Q9P275	UBP36_HUMAN	845	PHGKRKRKKKK
Q9P2D0	IBTK_HUMAN	979	MFKKAKTKAKK
Q9UIF9	BAZ2A_HUMAN	670	EVPKVKRGRGR
Q9UIG0	BAZ1B_HUMAN	895	GIAKAKLVMRR
Q9UK61	TASOR_HUMAN	105	FQIPRKSREKK
Q9UPP1	PHF8_HUMAN	523	LSLPSKNGSKK
Q9UPV0	CE164_HUMAN	114	IKKKKKKKEKK
Q9UPV0	CE164_HUMAN	111	SGAIKKKKKKK
Q9UQ35	SRRM2_HUMAN	193	KKKKKKKDRGR
Q9UQ35	SRRM2_HUMAN	1754	SSPRTKTTSRR
Q9UQR1	ZN148_HUMAN	394	KLVLKKINSKR
Q9Y2L1	RRP44_HUMAN	618	LNKLAKILKKR
Q9Y4C8	RBM19_HUMAN	823	TLARKKQVPRK
Q9Y620	RA54B_HUMAN	135	WCKPSKKKHKK
Q9Y6X0	SETBP_HUMAN	586	TPVKKKRGRPK
Q9Y6X0	SETBP_HUMAN	1016	SDLKSKKKRGR

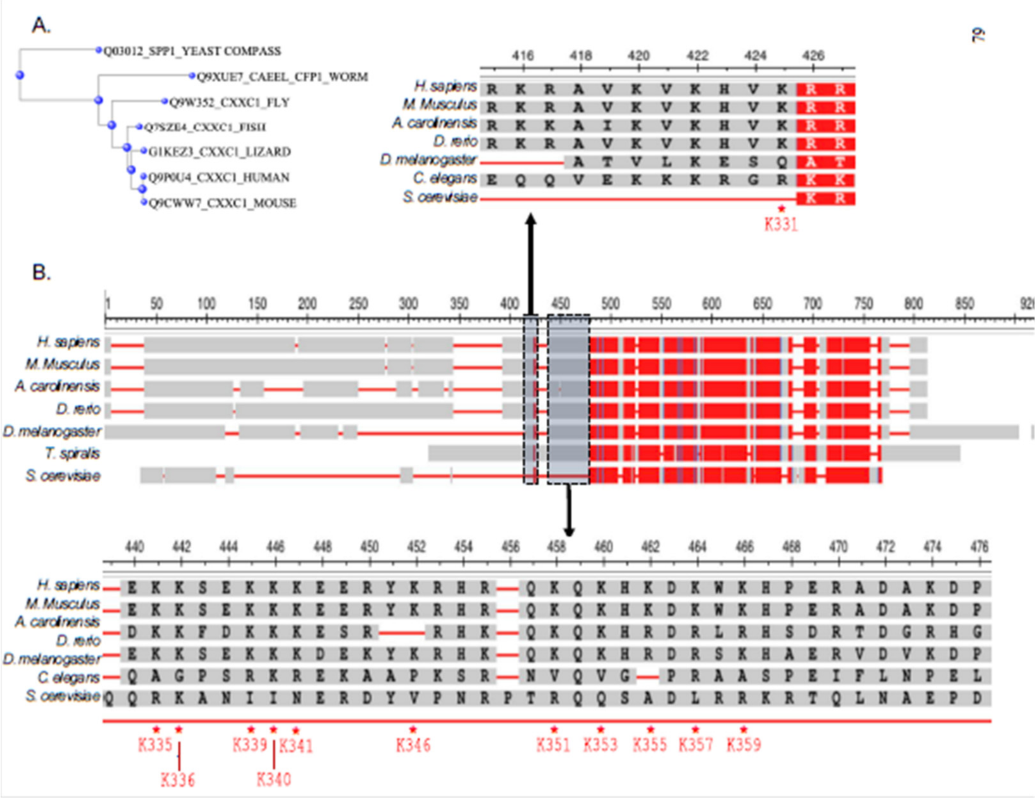

Supplemental Figure S1. (A) Cladogram illustrating the evolutionary relatedness of CFP1 in *H. sapiens* (Human), *M.Musculus* (Mouse) *A. carolinensis* (Lizard) *D. rerio* (Fish) *D. melanogaster* (Fly) *C. elegans* (Worm), *and S. cerevisiae* (Yeast). (B) Methylated lysines in the basic region of CFP1 are conserved.
